# Scale-adaptive surface modeling of vascular structures

**DOI:** 10.1186/1475-925X-9-75

**Published:** 2010-11-19

**Authors:** Jianhuang Wu, Mingqiang Wei, Yonghong Li, Xin Ma, Fucang Jia, Qingmao Hu

**Affiliations:** 1 Shenzhen Institutes of Advanced Technology, Chinese Academy of Sciences, Shenzhen, PR China; 2 The Chinese University of Hong Kong, Hong Kong SAR, PR China

## Abstract

**Background:**

The effective geometric modeling of vascular structures is crucial for diagnosis, therapy planning and medical education. These applications require good balance with respect to surface smoothness, surface accuracy, triangle quality and surface size.

**Methods:**

Our method first extracts the vascular boundary voxels from the segmentation result, and utilizes these voxels to build a three-dimensional (3D) point cloud whose normal vectors are estimated via covariance analysis. Then a 3D implicit indicator function is computed from the oriented 3D point cloud by solving a Poisson equation. Finally the vessel surface is generated by a proposed adaptive polygonization algorithm for explicit 3D visualization.

**Results:**

Experiments carried out on several typical vascular structures demonstrate that the presented method yields both a smooth morphologically correct and a topologically preserved two-manifold surface, which is scale-adaptive to the local curvature of the surface. Furthermore, the presented method produces fewer and better-shaped triangles with satisfactory surface quality and accuracy.

**Conclusions:**

Compared to other state-of-the-art approaches, our method reaches good balance in terms of smoothness, accuracy, triangle quality and surface size. The vessel surfaces produced by our method are suitable for applications such as computational fluid dynamics simulations and real-time virtual interventional surgery.

## Background

In surgical planning, treatment evaluation, and medical education, the geometric modeling of vascular structures is of vital importance. Three-dimensional (3D) models can help surgeons better understand the branching patterns and complex topology of vascular structures in a short time for better and quick decision making during surgery by providing straightforward information on the morphology of the vessels, the spatial relationships among these vessels and other relevant anatomic structures, and an intuitive depiction of curvature and depth relations [1-4].

The surface modeling techniques of vascular tree structures can be broadly classified as either model-based or model-free techniques [1]. Generally, the former methods require vessel centerline extraction and vessel diameter determination from segmented vessels [5]. Based on the centerline model (defined by the centerline and radius), geometric primitives such as cylinders [6] and truncated cones [7] are employed to fit the vessel surface for visualization. Unfortunately, the smoothness of the surface produced by these methods is poor, especially where the vessel branches. At these points, transition is unavoidably discontinuous and therefore has significant artifacts, resulting in very low visual quality. To achieve high-quality surface, other advanced surface representations have been investigated, such as, B-spline surfaces [8], simplex meshes [9], convolution surfaces [10], and subdivision surfaces [11,12]. Most of these methods yield desirable smooth surfaces; however, they suffer from low accuracy, badly shaped triangles or a large number of polygons.

The most common model-free technique of surface reconstruction in medical visualization is Marching Cubes (MC) [13]. Although effective in capturing overall shape, this technique has two major limitations. One limitation is that the generated surface heavily relies on the chosen isovalue and a slight change in value may result in great change in both the topological and geometrical features of the generated surface. The other limitation is that the visual quality is very low because the generated surface contains strong aliasing artifacts. Furthermore, these artifacts may lead to unstable numerical problems when the generated surface is applied for computational fluid dynamics (CFD) simulations. Recently, Schumann et al. [14,15] presented a model-free technique that could produce smooth surface from vessel segmentation. The technique is based on multi-level partition unity (MPU) implicits [16] originally dedicated to reconstruct the surface from 3D point clouds.

However, the main drawback of model-based methods is that their assumed models are unable to represent the underlying image data, and are therefore inappropriate for vessel diagnosis where high accuracy of surface representation is required. These methods assume that the cross-section of vessels is circular, whereas the pathologic vessels in clinical practice such as aneurysms might generally have a non-circular shape (e.g. ellipse) [2,5]. In contrast, model-free methods make no model assumptions and represent the underlying data with high fidelity. Therefore the reconstructed surface from model-free methods could be used for vessel diagnosis.

this paper, we present a model-free approach that relies on a prior vessel segmentation result, point extraction, Poisson equations and adaptive polygonization. With the proposed weight function, the triangulation algorithm in the gap-stitching stage can produce a two-manifold triangulation that maximizes the minimal angle of the triangle. Our approach yields a both morphologically correct and topologically preserved two-manifold smooth surface that is scale-adaptive to the local curvature of the surface by increasing/decreasing the size of triangles in regions with low/high curvatures. In addition, our method generates fewer and better-shaped triangles that are suitable for applications such as CFD computations and finite element analysis, and does not require other additional geometry processing techniques to improve triangle quality or to reduce number of triangles.

This paper is organized as follows. Details of our method are described in Sections Method. The results and discussion are presented in Section Results and Discussion. Finally, our conclusions are given in Section Conclusions.

## Methods

### Overview

**Figure 1:**
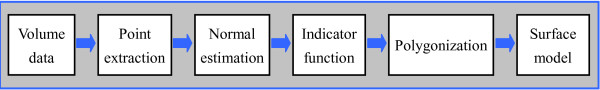
**Pipeline of the geometric modeling of vascular tree structures**. Pipeline of the geometric modeling of vascular tree structures

The pipeline of the presented approach to the geometric modeling of vascular tree structures is illustrated in Figure 1. The pipeline begins with a 3D binary volume data from segmented image (1 represents the voxels of the vessel structures and 0 represents the background). The boundary voxels between the segmented vessel and the background are extracted and used to build a 3D point cloud. Then, a 3D implicit indicator function is computed from the oriented 3D point cloud by solving a Poisson equation. Finally the surface model is generated by a proposed polygonization algorithm for explicit 3D visualization.

### Point extraction

**Figure 2:**
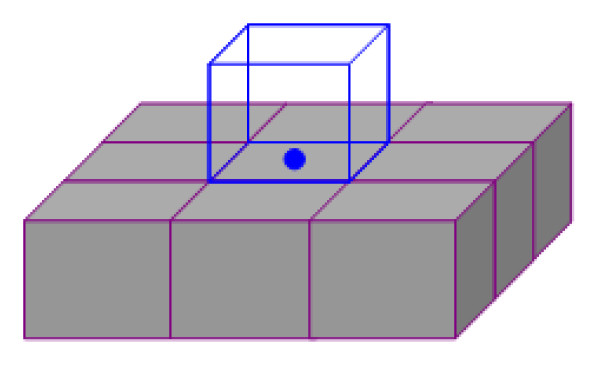
**Example of point extraction**. Example of generating point (blue) based on constellations of object voxels (grey) and outer boundary voxel (blue)

The point extraction step aims to faithfully represent the boundary of the vessel using 3D point clouds. Generally, point generation from volume data is driven by the voxel grid of the segmented result [14,17]. Braude et al. [17] used the boundary voxels to directly generate the point cloud; unfortunately their work failed to represent very thin vessel branch which might be represented as lines. To reconstruct the very thin vessel structures and to prevent aliasing artifacts, we use a similar adaptive point extraction technique [14,15] in our pipeline. The technique relies on the constellation of adjacent object voxels and outer boundary voxels that are closest to the given object voxels, e.g. if there is only one object voxel in the 3D-6-neighborhood, one point will be generated in the center of the boundary face, which is defined as the voxel adjacent to the object neighbor voxel, as illustrated in Figure 2. To sufficiently represent the thin vascular branch, voxels representing thin structures are first identified by a top-hat-transformation with a 3x3x3 structuring element, after which all outer boundary voxels adjacent to thin structures are refined into eight subvoxels [14].

### Normal vector estimation

To define a vector field for computing the indicator function, we need to estimate the normal vectors of the extracted point cloud. Generally, a normal vector is computed based on the image gradient of a given voxel in the segmentation result. However, this method may not be effective when the neighborhood of a voxel is symmetrical because the points are placed in the centers of the boundary faces. In this case, according to Schumann et al. [14,15], the normal of the face is taken as the normal of the generated point. This method is simple; however, it may achieve undesirable results when the voxel size is large. To avoid this problem and robustly estimate the normal vector for a given sample point, we use a method relying on covariance analysis [18]. The 3x3 covariance matrix **C **for a sample point *P *is defined as follows:

(1) $$C={\left[\begin{array}{c} p_{i,1}-\bar{p}\\ \cdots \\ p_{i,k}-\bar{p} \end{array}\right]}^{T}\cdot \left[\begin{array}{c} p_{i,1}-\bar{p}\\ \cdots \\ p_{i,k}-\bar{p} \end{array}\right]\bar{p}=\sum_{j=1}^{k}p_{i,j}$$

where {*p*_*i*,1_, *p*_*i*,2_,⋯, *p*_*i*, *k*_}is the *k nearest neighbors *of the point *P*. Since **C **is symmetric and positive semi-definite, all eigenvalues are real-valued and all eigenvectors form an orthogonal frame. The eigenvector corresponding to the smallest eigenvalue is taken as the normal vector of the point *P*.

### Indicator function

After obtaining the point clouds with oriented normals, many techniques are available to reconstruct them for surface visualization. For a comprehensive introduction to these techniques, please refer to a recent survey [19]. One popular surface reconstruction technique is the implicit function technique. This technique first constructs a 3D function that approximates/interpolates the point samples and then polygonizes the reconstructed surface. The main advantage of this type of technique is that it can reconstruct a watertight surface from different 3D models with any topological complexity. We choose the Poisson surface reconstruction [20] technique to model the vessel surface because, unlike the MPU technique [16], it is robust to recover fine details from noisy data and does not need to resort to heuristic partitioning or blending for surface fitting.

The basic idea behind Poisson surface reconstruction is to utilize the vector field $\vec{V}$ to compute the indicator function *ψ *(defined as 1 at points inside the surface and 0 at points outside). At points near the surface, the gradient of *ψ *is a vector field that is equal to the normal vector field. Hence, the problem of computing the *ψ *turns into finding a function whose gradient best approximates the $\vec{V}$, i.e. $\min_{\psi }||\nabla \psi -\vec{V}||$. After applying the divergence operator, the problem becomes a standard Poisson problem [20]:

(2) $$\Delta \psi \equiv \nabla \cdot \nabla \psi =\nabla \cdot \vec{V}$$

To reconstruct fine details, an adaptive octree ξ defined by the position of the sample points is used to represent the implicit function, and each node *O *∈ ξ of the tree is associated with a function *F_o _*when the following conditions are satisfied [20]: 1) the vector field can be expressed as the linear sum of the *F_o_*; 2) the matrix representation of the Poisson equation can be solved efficiently; and 3) the representation of the indicator function can be accurately evaluated near the surface. For every node *o *∈ ξ, the *F_o _*is set to be the unit-integral centered about the node *o *and scaled by the size of *o*:

(3) $$F_{o}(q)=F(\frac{q-c}{w})\frac{1}{w^{3}},F(q)=f(\frac{q}{2^{d}})$$

where *q *is the sample point; *c *and *w *are the center and width of the node *O*, respectively; *d *is the maximum tree depth; and *f *is a Gaussian filter with unit variance.

To allow for sub-node precision, the gradient field of the indication function is defined as follows:

(4) $$\vec{V}(q)=\sum_{s\in S}\sum_{o\in \Omega }\varpi F_{o}(q)\vec{n}$$

Where *S *is the input data with a set of samples *s *∈ *S*, each consisting of a point and an inward-facing normal $\vec{n}$; Ω is the set of eight depth *d *nodes closest to the sample point; and ϖ is a trilinear interpolation weight. After defining the vector field, the indicator function is obtained by solving the Poisson equation Eq. (2) using a conjugate gradient solver.

### Polygonization

To explicitly visualize the implicit surface, we need to triangulate the implicit surfaces from the computed indicator function. Our polygonization algorithm consists of two stages: mesh-expanding and gap-stitching.

### Mesh-expanding stage

**Figure 3:**
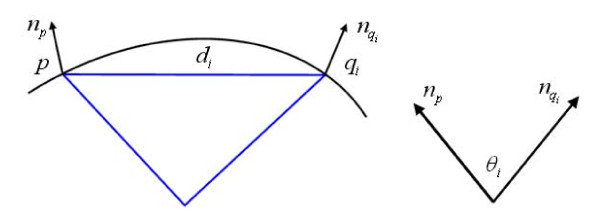
**Illustration of calculating the radius of curvature at a given point**. The illustration of calculating the radius of curvature at a given point

We first introduce how to compute the radius of the curvature of a given point and then describe the mesh-expanding procedure. We choose a point *x *near the surface, and compute its corresponding surface point *p*, and the surface normal at *p*, denoted by *n_p_*, using an iterative procedure known as Newton step [21]. The radius of curvature at point *p *is estimated by calculating the radius of curvature of several geodesics that cover *p*, and taking the minimum one [22,23]. Assuming a set of surface point *q_i _*with surface normal $n_{q_{i}}$, are close to the point *p*, let *d_i _*be the distance between *p *and *q_i_*, and *θ_i _*be the angle between *n_p _*and $n_{q_{i}}$, as illustrated in Figure 3. The radius of the curvature at point *p *is then calculated by

(5) $$r(p)=\min(\frac{d_{i}}{2\sin(\frac{\theta_{i}}{2})})$$

**Figure 4:**
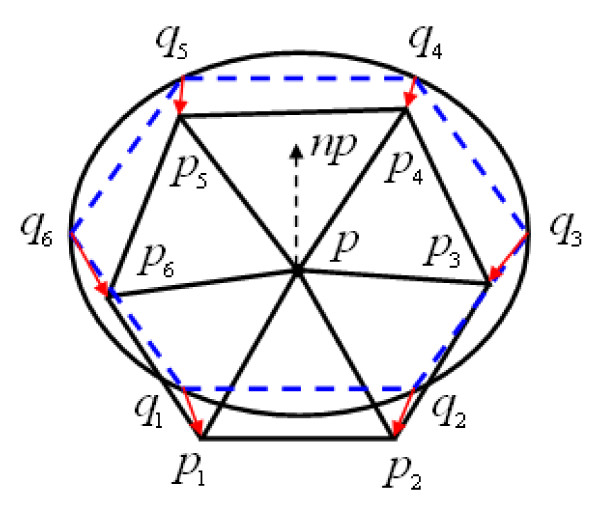
**The generation process of initial mesh**. First generating a regular hexagon (the dashed lines in blue) in the plane tangent to *p*, and projecting the vertices of the hexagon onto implicit surfaces to estimate curvature. Then adjusting the edge length of the hexagon to *ρ*·*r*, and finally, projecting the vertices of hexagon onto surface again to generate an initial mesh, consisting of vertices *p*_1_,⋯,*p*_6 _and *p*.

Starting from point *p*, we construct six triangles as an initial mesh. First, the point *p *is surrounded by constructing a regular hexagon *q*_1_, *q*_2_,⋯,*q*_6 _in the plane tangent to *p*. To adapt to the local curvatures of the surface, we project *q*_1_, *q*_2_,⋯,*q*_6 _onto the surface to estimate *r*, the radius of curvature at point *p*. Then, the generated regular hexagon is adjusted, whose edge lengths are *ρ*·*r*, where *ρ *is a user-defined constant. Finally, the vertices of the hexagon are again projected onto the surface, and denoted as *p*_1_, *p*_2_,⋯,*p*_6_. The triangles formed by the hexagon *p*_1_, *p*_2_,⋯,*p*_6 _with *p *are the first six triangles of the mesh (see Figure 4). This mesh is considered as the seed element and is expanded by progressively growing triangles from its boundary edges.

**Figure 5:**
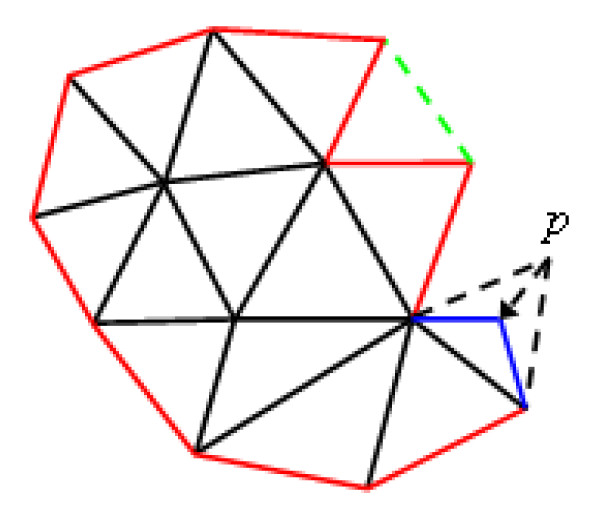
**A fragment illustrating the mesh-expanding procedure**. The red lines are boundary edges of the mesh and stored in a queue. From the boundary edges, new triangles are progressively generated. Note that the blue lines combined with one boundary edge will construct a new triangle if it satisfies two specified conditions. If two boundary edges make an angle less than 70 degrees, the three vertices on these two edges are used to produce a new triangle, see the triangle consisting of the green dashed line and the other two boundary edges.

Once the initial mesh is generated, its boundary edges are placed into a queue. We then take an edge, denoted as (*u*, *v*), from the queue. A new point *p *is created if *u*, *v *and *p *form an equilateral or close-to-equilateral triangle. The new triangle Δ*uvp *is coplanar and opposite to the existing triangle containing the edge (*u*, *v*). After point *p *is placed onto the implicit surfaces to estimate curvature, it is changed on the original plane such that the lengths of the edges (*p*, *u*) and (*p*, *v*) are equal to*ρ*·*r*. Finally, the *p *is once again placed onto the implicit surfaces (Figure 5).

The newly constructed triangle Δ*uvp *is added to the mesh if it satisfies two conditions. One is that both edge (*p*, *u*) and edge (*p*, *v*) should make an angle of at least 50 degrees (the maximal angle is 70 degrees) with the edge (*u*, *v*) in the old mesh. The other condition is that the triangle Δ*uvp *should not approach existing triangles too closely. These two conditions guarantee that the resulting triangles are close-to-equilateral and the gap generated by this stage is not too narrow to sew in the subsequent gap-stitching stage. If any one of the boundary triangle in the mesh (denoted as *T*) is closer to triangle Δ*uvp *than one-third of the length of the longest edge in *T *and triangle Δ*uvp*, then the triangle Δ*uvp *is not added to the mesh. Otherwise, the triangle Δ*uvp *is added to the mesh and the boundary edges (*p*, *u*) and (*p*, *v*) are placed into the queue. The mesh-expanding terminates when the queue is empty.

### Gap-stitching stage

**Figure 6:**
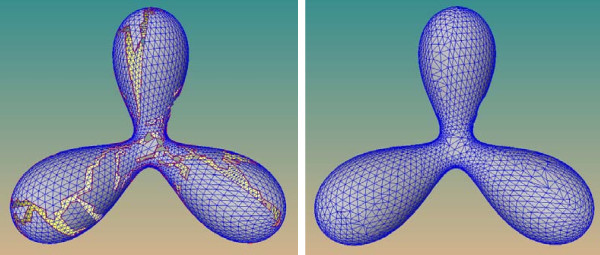
**Polygonization of a trifurcate model**. A long gap is produced upon the termination of mesh expanding stage (left), and is sewed in the subsequent gap-stitching stage (right).

To stitch the gap produced in the mesh-expanding stage, as shown in Figure 6 (left), it should first be identified. Since the gap is a closed loop of boundary edges in the mesh, and the mesh produced after mesh-expanding stage is a connected two-manifold surface, the gap can be stitched as described below.

Let Φ(*i*, *j*, *k*) be a weight function defined on the set of all triangles (*p_i_*, *p_j_*, *p_k_*) that could be possibly generated in triangulating the polygon *p_i_*,⋯, *p_j_*, 0 ≤ *i *<*j *<*n *and let *w*_*i*, *j *_be the minimum total weight that can be achieved during the triangulation process. Then the triangulation algorithm proceeds as follows:

i. For *i *= 0, 1,⋯, *n *- 2, let *w*_*i*, *i*+1 _= 0, and for *i *= 0,1,⋯,*n *- 3, let *w*_*i*, *i*+2_: = Φ(*i*, *i*+1, *i*+2). Set *j*: = 2.

ii. Put *j*: = *j*+1. For *i *= 0,1,⋯,*n - j - *1 and *k *= i + *j*. Let $w_{i,k}:=\underset{i<m<k}{\min}[w_{i,m}+w_{m,k}+\Phi (v_{i},v_{m},v_{k})]$. Let *O*_*i*, *k *_be the index *m *where the minimum is achieved.

iii. If *j *<*n *- 1, then return to step 2; otherwise the weight of the minimal triangulation is *w*_0,*n*-1_.

iv. Let Θ: = *φ *and call the recursive function *Trace *with the parameters (0, *n*- 1).

Function *Trace *(*i*, *k*):

If (*i *+ 2) = *k*, then Θ: = Θ∪Δ*v*_*i*_*v*_*i*+1_*v*_*k*_;

Else

1) Let *o*: = *O*_*i*, *k*_;

2) If *o *≠ *i *+ 1, then *Trace *(*i*, *o*);

3) Θ: = Θ∪Δ*v_i _v_o _v_k_*;

4) If *o *≠ *k *- 1, then *Trace *(*o*, *k*)

End else.

At the end of the algorithm, Θ contains the required triangulation of polygon *p*_0_,⋯,*p*_*n*-1_. The triangulating steps are similar to the schemes described in [24,25], but different in defining the weight function Φ(*i*, *j*, *k*). Barequet and Sharir [24] suggested the function as the area of triangle (*i*, *j*, *k*), whereas Liepa [25] designed it by combining the dihedral angles between the neighboring triangles with areas of triangles. However, when the holes are highly irregular, the resulting mesh may not be a topologically preserved two-manifold surface. To avoid this, we propose a strategy wherein the angles of potential triangle (*p_i_*, *p_k_*, *p_j_*), *i *<*k *<*j*, denoted as *T*, are first taken into account, with the minimal angle of triangle *T *being maximized. The dihedral angles and area are then considered the same time. Therefore, we define triples as follows:

(6) Φ(i,k,j)=(α,β,A)

where *α *is the maximized minimal angle of triangle *T*, *β *is the maximal dihedral angle between triangle *T *and its neighborhoods, and *A *is the area of triangle *T*. The ordering in *Φ *is designed to give precedence to *α *over *β*, and *β *over *A*:

(7) (α1,β1,A1) <(α2,β2,A2):⇔((α1 >α2)∨(α1=α2∧β1 <β2)∨(α1=α2∧β1=β2∧A1 <A2))

The addition operator sums the area but retains the maximized minimal angle and the "worst" (i.e., largest) dihedral angle:

(8) $$\left(\alpha_{1},\beta_{1},A_{1})+(\alpha_{2},\beta_{2},A_{2}):=(m_{1},m_{2},A_{1}+A_{2}\right)$$

where *m*_1 _= min(*β*_1_, *β*_2_), and *m*_2 _= max(*β*_1_, *β*_2_).

With our weighting function, the triangulation algorithm can produce a two-manifold triangulation that maximizes the minimal angle of triangle *T*. After triangulation, the patching triangles are subdivided to make their density similar to the density of surrounding mesh [25,26]. An example of gap stitching is illustrated in Figure 6 (right).

## Results and Discussion

**Figure 7:**
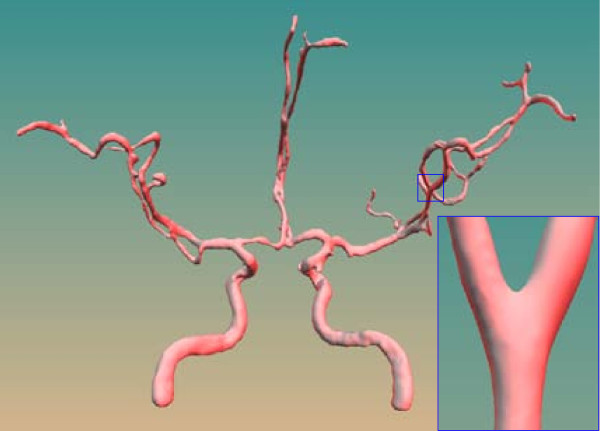
**A cerebral vessel surface model produced by our approach**. A cerebral vessel surface model produced by our approach

**Figure 8:**
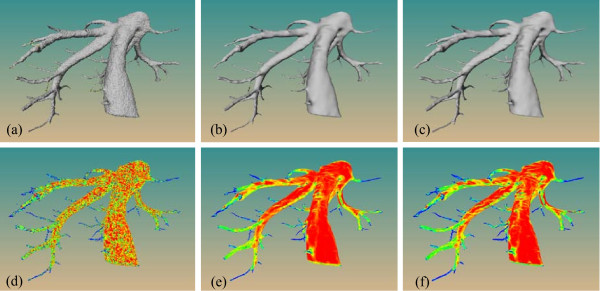
**Comparison of the geometric modeling results on a liver tree**. Surface model generated by the MC algorithm (a), MPU-based algorithm (b) and our approach (c). Color-coded visualization of the root mean square curvature distribution for the generated surface using MC algorithm (d), MPU-based algorithm (e) and our approach (f).

**Figure 9:**
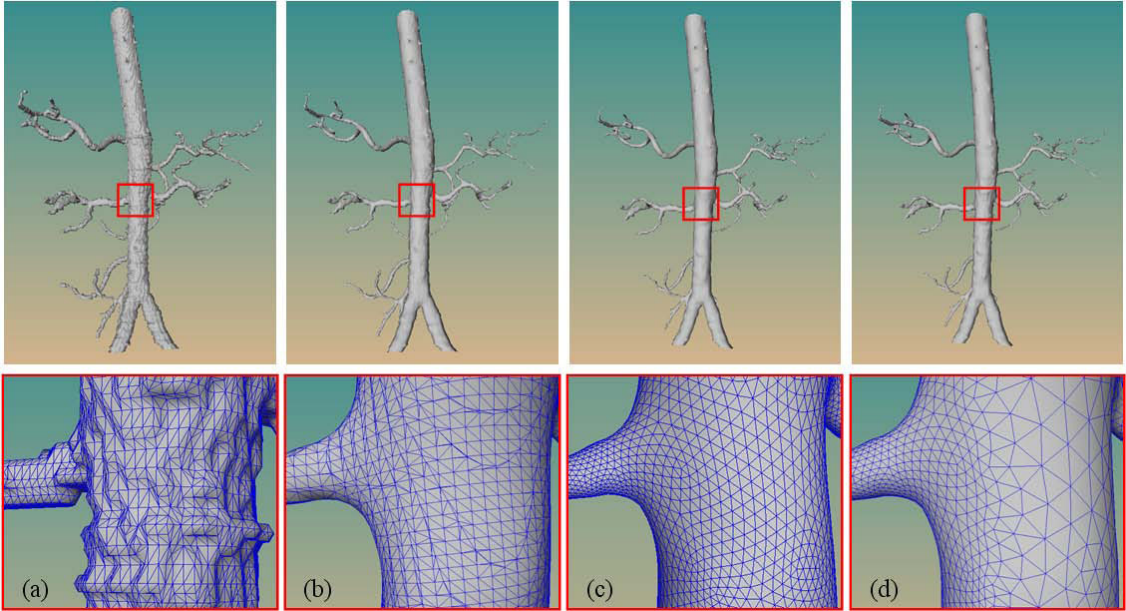
**Comparison of triangle quality for an aorta tree**. Surface model generated by the MC (a), MPU-based method (b), SS-based method (c) and our method (d). The bottom row is a zoomed region corresponding to the rectangle region of the top row.

We have applied the presented model-free approach to the geometric modeling of a variety of vascular trees, namely, cerebral (Figure 7), liver (Figure 8) and aorta trees (Figure 9). The binary segmentation results of the liver and cerebral tree are from our manual segmentation whereas the aorta tree is from the public resource (http://www.ircad.fr). Table 1 summarizes the properties of the data tested in this paper. The produced surfaces, as shown in Figure 7, are smooth, especially at transition, and do not show aliasing artifacts. A close visualization shows that the morphology of vascular structures and thin structures (even the thin elongated structures) can be reconstructed with the correct topology.

**Table 1:** Summary of properties of the data sets Sample table title

Dataset	Modality	Resolution	Voxel size
Liver tree	CT	512 × 512 × 279	0.6563 × 0.6563 × 0.5

Cerebral tree	MRA	512 × 512 × 170	0.51 × 0.51 × 0.80

Aorta tree	CT	512 × 512 × 167	0.961 × 0.961 × 1.80

We evaluated our method in terms of surface smoothness, surface accuracy, triangle quality, surface size and efficiency on the tested dataset. We also compared our approach with the conventional model-free algorithm, i.e. MC algorithm, and state-of-the-art algorithms, i.e. model-free MPU-based algorithms (MPU-based) [14,15], and model-based subdivision surface algorithm (SS-based) [11,12]. In our experiments, the implementation of SS-based is slightly different than in [11,12]. In [11,12], after obtaining an initial mesh from the centerline model, the Catmull-Clark scheme [27] is applied to generate the vessel surface; thus the surface is a quadrilateral mesh. Since the surfaces produced by MC, MPU-based and our method are all triangular meshes, for the convenience of comparison, we applied the Loop scheme [27] with three iterations to produce vessel surface (for regular meshes, the surfaces yielded by the Loop scheme and Catmull-Clark scheme are both C^2^-continuity [27]). For the MPU-based algorithm, we attempted to select parameter settings suggested in [14,15] with the best results. In our approach the user-defined *ρ *is set to 0.15 in the mesh-expanding stage and the parameters * k *is set to 10 in the normal vector estimation stage.

### Surface smoothness

We compared our approach with MC and MPU-based algorithm when applied to the same segmentation result. As illustrated in Figure 8, the surface produced by the MC (Figure 8a) has a visually low surface quality and contains a great variety of artifacts, which might disturb the visual interpretation of the vessel surface and therefore affect decision making during diagnosis. In contrast, the surfaces generated by both the MPU-based (Figure 8b) and our approach (Figure 8c) are highly smooth.

To validate the smoothness of the surface, we computed the distribution of the curvature value on the surface. The curvature map can directly help us observe the flaws and roughness of the surface that are not easily identified by human eyes. Here, we computed the root mean square (RMS) curvature of both the maximal *k*_max _and the minimal *k*_min _principal curvatures respectively, and RMS is defined as $\sqrt{(k^{2}{}_{\max}+k^{2}{}_{\min})/2}$. The principal curvatures are computed based on a finite-differences technique [28].

Figure 8d, Figure 8e, and Figure 8f show the curvature distribution of the MC surface, MPU-based surface and the surface generated by our method respectively. It can be seen that due to the substantial staircase artifacts, the curvature distribution of the MC surface is highly inhomogeneous compared to that of both the MPU-based method and our method. For the thin vessel structures, the distributions of other methods are similar to that of the MC surface. The difference of surface smoothness between our approach and MPU-based algorithm is not very apparent. The reason is that these two approaches both utilize an implicit descriptor as the underlying surface representation. Although the smoothness of the MC surface can be improved with additional geometry processing techniques, e.g. smoothing filter, this unfortunately leads to volume shrinkage, collapse of thin vessel structures and unfaithful representation of the underlying data [29].

### 3.2 Surface accuracy

We analyzed the accuracy of the generated surface on the assumption that the input binary segmentation result of our pipeline had been validated correctly. To provide a quantitative comparison of the surfaces, we measured the distance error between two surfaces using the *MESH *tool [30]. The tool utilizes Hausdorff distance to calculate the maximum, mean and RMS errors between two specified surfaces. In this experiment, the surface generated by the MC algorithm is taken as reference surface (although it is not the most accurate technique to visualize the segmentation result, it has been the de facto standard in medical surface visualization and has been widely applied in numerous radiological workstations [5,15]).

Table 2 lists the mean, maximum and RMS errors for the tested dataset. The maximum error of SS-based method is larger than twice a voxel size, and is also larger than that of both the MPU-based and our method, whose maximum error is less than a voxel size. The reason is that, aside from the simplified model assumptions of circular cross-sections, approximating subdivision scheme leads to volume shrinkage for a closed surface during its convergence to the limit surface. Due to the same point extraction strategy, the errors between MPU-based and our method are very similar. However, the maximum error remains larger than half of a voxel size. This occurs in the feature regions of the vessel surface, such as small concave and convex regions, which are not represented by sufficient points, even though an adaptive subsampling technique is applied [14,15].

**Table 2:** Accuracy of MPU-based method, subdivision surface-based method and our method for the tested dataset Mean, max and RMS denote mean distance error, maximum distance error and root mean square distance error.

Dataset	MPU-based			SS-based			Our method		
	
	mean	max	RMS	mean	max	RMS	mean	max	RMS
Liver tree	0.098	0.321	0.016	-	-	-	0.098	0.320	0.115

Cerebral tree	0.084	0.512	0.106	0.171	1.782	0.213	0.082	0.510	0.104

Aorta tree	0.225	1.635	0.373	0.512	3.776	0.892	0.226	1.635	0.374

### Triangle quality

Like surface accuracy, triangle quality is an important factor in achieving accurate results in many simulations. CFD simulations require the input surface to be free from block and staircase artifacts; therefore, the triangle meshes should have a good quality with regard to edge ratio [31]. Degenerated triangles such as thin and elongated triangles may lead to numerical unstability in CFD simulation, and may even make the simulation impossible. Smooth transition at the points where the vessel surface branches is also a prerequisite. Furthermore, the triangle size should not change abruptly, and surface regions with high curvature are desirably represented by small triangles.

Figure 9 demonstrates the comparison of triangle quality for the aorta tree. It can be seen once again from the zoomed region that the MC surface contains strong staircase artifacts and badly shaped triangles, and is not naturally smooth at the transition of the branches. The surface generated by the MPU-based method has a smooth transition, but also contains degenerated triangles. Although these triangles can be removed by invoking additional mesh quality improvement techniques [15], special care must be taken to preserve vital surface features during the optimization process. Because of the good underlying property of subdivision surface, the surface produced by the SS-based method is composed of well-shaped triangles, with smooth transition at the branches. However, the surface accuracy of the SS-based method is low (see Section Surface size) and unsuitable for CFD simulations [31]. Similar to the SS-based method, the surface of our method also has a smooth transition without badly shaped triangles. Additionally, the triangle size yielded by our method is adaptively scaled to the local differential geometric surface characteristics. The surface areas with high curvature are represented by smaller triangles, whereas triangles become large in the relative low-curvature region. Meanwhile, the triangle size from small to large is changed gradually.

**Figure 10:**
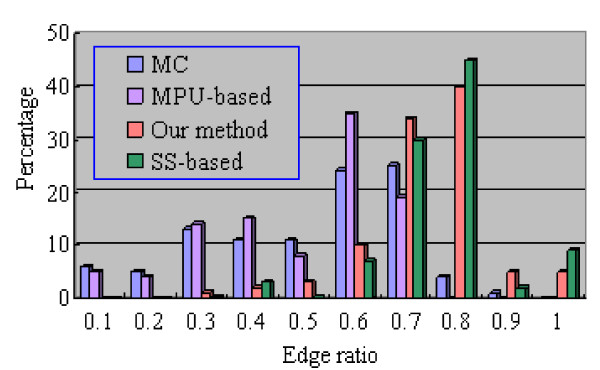
**The distribution of edge ratio of the aorta tree**. The distribution of edge ratio of the aorta tree.

*Edge ratio *is one measure for triangle quality, and is defined as *τ *= |*t*|_0_/|*t*|_∞_, where |*t*|_0 _is the minimum edge length of a given triangle, and |*t*|_∞_is the maximum length [32]. It is straightforward to see that *τ *≤ 1, whereas the equality occurs when the triangle is equilateral, and *τ *converges to zero if the triangle is highly needle-shaped. An ideal triangular mesh has triangles that are all equilateral, and should adapt to local surface properties, such as curvature. Figure 10 shows the distributions of edge ratio for aorta tree. It can be seen that the MC and MPU-based methods yield approximately 5% very badly shaped triangles (*τ *< 0.1) such as thin elongated triangles, and less than 6% well-shaped triangles (*τ *≥ 0.8). Moreover, these two methods suffer from enormous standard deviations of ratio. In contrast, approximately 50% of generated triangles (*τ *≥ 0.8) by SS-based and our methods are close-to-equilateral. Unsurprisingly, the two latter methods with small standard deviation yield no thin elongated triangles. However, our method produces some triangles (less than 1%) with relative small ratio (*τ *= 0.3). These triangles are constructed to stitch the gap in the polygonization.

### Surface size

Here surface size refers to the total number of triangles and vertices approximating a surface. Surface size affects surface accuracy, surface rendering speed, and human interactive response. In the MPU-based method, Bloomental's implicit polygonizer [33] is applied to produce a triangular mesh. In the polygonization, if the grid size is set too large, the size of generated triangle is also very large, resulting in a loss of finer details such as thin vessel structures. If the grid size is too small, the polygonization process will be time-consuming; the surface size will be huge and will slow down interactive rendering frame rates. Mesh simplification techniques are therefore usually invoked as a subsequent step to reduce the surface size. However, this step may lead to a loss of both topological and geometrical features, and may even produce degenerated triangles during the simplifying process. In the SS-based method, the smoothness of the surface is increased with the iteration of subdivision; unfortunately, the number of polygons grows exponentially. Each iteration of subdivision yields a three-time increase in polygon due to the underlying topological refinement rules. In our method, the size of triangle can be adapted to the local curvature of the surface. This feature can save many triangles in representations. In actuality, using many small triangles to represent surface regions with low curvature, such as a flat region, does not significantly improve surface smoothness but increases surface size.

Table 3 reports several statistics for the surface sizes of the three tested data. Compared with the MC and MPU-based method, our method generally saves approximately 20% and 10% in the number of triangles, respectively, whereas for a vessel tree consisting of many highly curved branchings such as cerebral tree, the saving is more than 30% and 20% respectively. The surface size produced by the SS-based method at third iteration is smaller; however, it will be greatly larger at fourth iteration than that of MC and MPU-based method. Taking the cerebral tree as an example, the number of vertices and triangles after four iterations of subdivision are up to 102443 and 204976, resulting in triangles smaller than the original voxel.

**Table 3:** Surface sizes for three vessel surfaces generated by the MC, MPU-based method, SS-based method and our method

Dataset	MC		MPU-based		SS-based		Our method	
	
	vertex	triangle	vertex	triangle	vertex	triangle	vertex	triangle
Liver tree	95920	191920	84404	168888	-	-	76728	153536

Cerebral tree	33788	67685	29505	59120	25567	51244	23347	46706

Aorta tree	48869	97520	44484	88753	40452	80604	34173	68163

### Computational efficiency

Our method utilizes implicit function to describe vascular structures; therefore, it requires an evaluator for the indicator function defined at all extracted points in space. The function is obtained by solving Poisson equations using the efficient linear solvers [34]. The computational cost mainly depends on the complexity and the resolution of input objects. In the mesh-expanding stage, the time is largely spent on calculating curvature radius and movement of points onto surface. However, in the gap-stitching stage, due to the *O*(*n*^3^) performance complexity of the triangulation algorithm, the time for this stage comprises approximately one-fifth of the entire time. However, the proposed approach is much slower when compared to the MC, MPU-based or SS-based method. Taking the cerebral tree, the most complex tested dataset, as an example, the overall time for our method is 127 seconds, whereas the MC only requires 5 seconds. The performances of this data for the MPU-based method and SS-based method are 53 and 66 seconds respectively.

## Conclusions

We have presented a model-free method for the geometric modeling of vascular structures. Our method yields both a topologically correct two-manifold and a geometrically smooth vessel surface. An important feature of the presented method is that it produces a surface that is scale-adaptive to the local curvature of the surface. This minimizes the number of triangles in the representation, leading to faster interactive rendering frame rates and saving much computational time in post-processing procedures, such as real-time blood flow simulations and collision detections in virtual interventional surgery.

We validated our method to a variety of vascular structures and compared the results with other state-of-the-art techniques, both model-based techniques and model-free techniques, in terms of surface smoothness, surface accuracy, triangle quality, surface size and efficiency. Compared to the MC and MPU-based methods, the surface generated by our method achieves comparable accuracy; however, it is more suitable for applications that require high-quality triangulations such as CFD computations and finite element analysis, because our method yields smaller surface size, better-shaped triangles and no thin elongated triangles. Therefore, invoking additional geometry processing techniques to improve mesh quality or to reduce surface size is not necessary for the presented method. Mode-based methods, such as the SS-based method, can produce smooth surface and well-shaped triangles. The simple circular model assumption results in a low accuracy that is inappropriate for vessel diagnosis or CFD simulations, but can be used for certain situations where accuracy is not very important, such as in medical educations. Fortunately, very recent work [35] showed that with an elliptical model assumption, the surface accuracy of SS-based method could be improved. The experimental results demonstrate that our method reaches a better balance with regard to surface accuracy, surface smoothness, triangle quality and surface size.

The investigation of computational efficiency has revealed the limitation of our method in its current implementation. Fortunately, implementing the time-consuming steps, such as the triangulation step on CUDA, a parallel computing engine developed by NVIDIA [36], seems to be a promising solution to the limitation and might be part of future work. Although the presented method makes no model assumption and achieves high accuracy, it does not imply that our method can be directly applied to diagnostic tasks, because our pipeline takes the binary segmentation result as input, and supposes that the segmentation is validated correctly. Therefore, combining the validation of input data with the pipeline as a preprocessing step is also planned for future studies.

## Competing interests

The authors declare that they have no competing interests.

## Authors' contributions

JW designed the study, performed the experiments and was responsible for the data analysis. MW and YL contributed to the implementation of the algorithm. MW, XM, and FJ contributed to the discussion and the suggestion through this project. QH contributed to the discussion and, along with JW, drafted the manuscript. All authors have read and approved the final manuscript.
